# *Psoralea corylifolia* extract induces vasodilation in rat arteries through both endothelium-dependent and -independent mechanisms involving inhibition of TRPC3 channel activity and elaboration of prostaglandin

**DOI:** 10.1080/13880209.2017.1383484

**Published:** 2017-10-05

**Authors:** Addis Kassahun Gebremeskel, Tharaka Darshana Wijerathne, Ji Hyun Kim, Min Ji Kim, Chang-Seob Seo, Hyeun-Kyoo Shin, Kyu Pil Lee

**Affiliations:** a Laboratory of Physiology, College of Veterinary Medicine, Chungnam National University, Daejeon, Republic of Korea;; b Basic Herbal Research Group, Korea Institute of Oriental Medicine, Daejeon, South Korea

**Keywords:** Ethanol extract, hypotensive, nitric oxide, COX, non-selective cation channel

## Abstract

**Context:** Fructus Psoralea, *Psoralea corylifolia* L. (Leguminosae), has been widely used in traditional medicines for the treatment of dermatitis, leukoderma, asthma and osteoporosis.

**Objectives:** In this study, we sought to study mechanisms underlying the vasoactive properties of *Psoralea corylifolia* extract (PCE) and its active ingredients.

**Materials and methods:** To study mechanisms underlying the vasoactive properties of PCE prepared by extracting dried seeds of *Psoralea corylifolia* with 70% ethanol, isometric tension recordings of rat aortic rings and the ionic currents through TRPC3 (transient receptor potential canonical 3) channels were measured with the cumulative concentration (10–600 μg/mL) of PCE or its constituents.

**Results:** Cumulative treatment with PCE caused the relaxation of pre-contracted aortic rings in the presence and absence of endothelium with EC_50_ values of 61.27 ± 3.11 and 211.13 ± 18.74 μg/mL, respectively. Pretreatment with inhibitors of nitric oxide (NO) synthase, guanylate cyclase, or cyclooxygenase and pyrazole 3, a selective TRPC3 channel blocker, significantly decreased PCE-induced vasorelaxation (*p* < 0.01). The PCE constituents, bakuchiol, isobavachalcone, isopsoralen and psoralen, inhibited hTRPC3 currents (inhibited by 40.6 ± 2.7, 27.1 ± 7.9, 35.1 ± 4.8 and 47.4 ± 3.9%, respectively). Furthermore, these constituents significantly relaxed pre-contracted aortic rings (EC_50_ 128.9, 4.5, 32.1 and 114.9 μg/mL, respectively).

**Discussion and conclusions:** Taken together, our data indicate that the vasodilatory actions of PCE are dependent on endothelial NO/cGMP and also involved in prostaglandin production. PCE and its active constituents, bakuchiol, isobavachalcone, isopsoralen and psoralen, caused dose-dependent inhibition of TRPC3 channels, indicating that those ingredients attenuate Phe-induced vasoconstriction.

## Introduction

Fruit of *Psoralea corylifolia* L. (Leguminosae), Bu Gu Zhi, a well-known traditional medicinal plant, has been widely used for many years in Asian medicine for the treatment of various diseases, such as psoriasis, leukoderma, asthma and osteoporosis (Chopra et al. 2013). Phytochemical studies have shown that the plant contains a variety of chemical compounds, including psoralenoside, psoralen, isopsoralen, bakuchiol, bakuchicin and isobavachalcone, among others (Tan et al. 2015). These chemical components have multiple biological properties, including antimicrobial, estrogenic, antitumor, osteoblastic and anti-inflammatory activities. Of the constituents, only bakuchicin has been proposed to possess relaxing activity in rat arterial segments (Cho et al. 2001; Li et al. 2011). However, the mechanism underlying the relaxation-promoting effect of *Psoralea corylifolia* extract (PCE) on arterial smooth muscle has never been reported.

Hypertension is the most common cardiovascular disease, which is the leading cause of death worldwide. Increased sodium-retaining hormones and sympathetic nervous system activity, overproduction of endothelium-derived contracting factors, and deficiencies of vasodilators such as nitric oxide (NO) are among the pathophysiological factors implicated in hypertension (Lind et al. 2000; Versari et al. 2009). Although many drugs that modulate these hypertension risk factors are currently on the market (Tamargo et al. 2015), a number of research groups have continued phytochemical studies designed to isolate and develop cardiovascular modulators from plant sources.

Under physiological conditions, the endothelium controls the tone of the underlying vascular smooth muscle, mainly through the production of a number of endogenous vasodilatory mediators, including NO, prostaglandins and a family of endothelial-derived hyperpolarizing factors (EDHFs) (Oparil et al. 2003). It is well established that calcium (Ca^2+^) entry in endothelial cell through nonselective cation channels, possibly members of the transient receptor potential (TRP) channel family, regulates endothelial intracellular Ca^2+^, thereby modulating NO synthase (Brayden et al. 2008). Ca^2+^-permeable ion channels expressed in vascular smooth muscle cells also regulate the membrane potential, intracellular Ca^2+^ concentration, and contractility of smooth muscle. Activation of transient receptor potential canonical 3 (TRPC3) and TRPC6 channels, members of the classic (or canonical) TRP subfamily, by stimulation of several excitatory receptors has been shown to cause myocyte depolarization, which stimulates a global increase in intracellular Ca^2+^ through activation of L-type voltage-dependent Ca^2+^ channels (VDCCs), which leads to vasoconstriction (Zhang and Gutterman 2011; Gao et al. 2012; Kochukov et al. 2013) – a process termed excitation-contraction coupling.

Depolarization-dependent influx of Ca^2+^ in myocytes is crucial for vascular smooth muscle contraction, and elevated endothelial cell Ca^2+^ is involved in the elaboration of endothelium-derived factors, processes that are disrupted in hypertension. Thus, considering the popular uses of herbal compounds in the treatment of hypertension, we here sought to elucidate the mechanisms underlying the vasoactive properties of PCE. Specifically, we used wire myography and electrophysiological techniques to investigate the effects of PCE on aortic rings isolated from rats and on human TRPC3 (hTRPC3) channels heterologously expressed in HEK293 cells, respectively.

## Materials and methods

### Preparation of PCE

**Figure 1.**
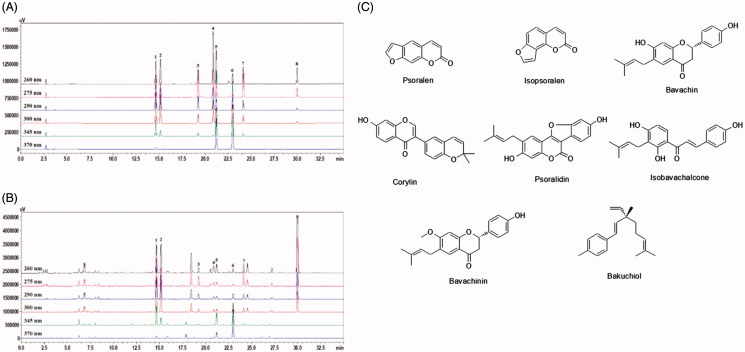
(A, B) HPLC chromatograms of the standard solution (A) and 70% ethanol extract of *P. corylifolia* (B). (C) Chemical structure of the eight marker compounds of PCE: psoralen (1), isopsoralen (2), bavachin (3), corylin (4), psoralidin (5), isobavachalcone (6), bavachinin (7) and bakuchiol (8).

Dried seeds of *P. corylifolia* were purchased from a local herbal store (Kwangmyungdang, Ulsan, Korea) in June 2011; their identity was confirmed by Professor Je-Hyun Lee (Herbology, Dongguk University, Gyeongju, Republic of Korea). A voucher specimen (2011-EBM-64) has been deposited at the K-herb Research Center, Korea Institute of Oriental Medicine. The dried seed (semen) of *P. corylifolia* (700 g) was extracted three times with 70% ethanol (7 L) for 60 min each using a Model 8510E-DTH ultra-sonicator (Branson, Danbury, CT). The extracted solution was then filtered through Whatman filter paper No. 2 (150 mm Ø; Maidstone, Kent, UK), evaporated to dryness using a Büchi Rotavapor R-210 (Büchi Labortechnik AG, Flawil, Switzerland), and freeze-dried (FD-5525L; Ilshin BioBase, Dongducheon, Korea) to give a powder. The ethanol extract yield was 102.5 g (14.6%). The eight reference standards, psoralen (purity 98.9%), isopsoralen (purity 99.8%), bavachin (purity 99.4%), corylin (purity 99.8%), psoralidin (purity 99.6%), isobavachalcone (purity 99.0%), bavachinin (purity 99.3%) and bakuchiol (purity 98.5%), were purchased from Shanghai Sunny Biotech (Shanghai, China). The chemical structures of the reference compounds are shown in Figure 1(C). HPLC-grade methanol, acetonitrile and water were obtained from J.T. Baker (Phillipsburg, NJ). Analytical-grade formic acid and dimethyl sulphoxide (DMSO) were obtained from Sigma-Aldrich (St. Louis, MO).

### High-performance liquid chromatography (HPLC) analysis

The eight marker components in *P. corylifolia* were quantitatively analysed by HPLC using a Shimadzu Prominence LC-20A Series instrument (Kyoto, Japan) equipped with a solvent-delivery unit (LC-20AT), online degasser (DGU-20A_3_), column oven (CTO-20A), auto sample injector (SIL-20AC) and photodiode array (PDA) detector (SPD-M20A). Data acquisition and processing were accomplished using Shimadzu LabSolution software (Version 5.54 SP3, Kyoto, Japan). All separation analyses were performed on a Waters SunFire C_18_ column (250 × 4.6 mm, 5 μm; Milford, MA) at 40 °C using a mobile phase of 0.1% (v/v) aqueous formic acid (A) and 0.1% (v/v) formic acid in acetonitrile (B) with gradient elution, as follows: 0–30 min, 30–100% B; 30–35 min, 100% B; 35–40 min, 100–30% B; 40–50 min, 30% B. The flow rate of the mobile phase was kept at 1.0 mL/min, and the injection volume was 10 μL. For quantitative analyses of the eight compounds (psoralen, isopsoralen, bavachin, corylin, psoralidin, isobavachalcone, bavachinin and bakuchiol) in *P. corylifolia*, a lyophilized sample (100 mg) of each compound was dissolved in 70% methanol (100 mL) and sonicated for 20 min. For bakuchiol, the solution was subsequently diluted 10-fold. All test solutions were filtered through a 0.2 μm membrane filter (PALL Life Sciences, Ann Arbor, MI) before HPLC injection.

### Animals

In this study, 250–300 g, male Sprague-Dawley rats obtained from Narabiotech Co. (Seoul, Korea) were used. The rats were kept in cages at room temperature (25 °C) and provided *ad libitum* access to standard rat diet and water. The animal care and use protocol was revised and approved by the Ethics Committee of the college of Veterinary Medicine, Chungnam National University (CNU-00222).

### Tissue preparation

Before each experiment, rats were sacrificed by cervical dislocation following anaesthesia using carbon dioxide. The thoracic cavity was immediately opened by midline laparotomy, and an aortic segment was gently dissected and placed in physiological saline solution (PSS) consisting of 120 mM NaCl, 2.5 mM CaCl_2_, 1.2 mM MgCl_2_, 11 mM glucose, 25 mM NaHCO_3_, 5.9 mM KCl and 1.2 mM NaH_2_PO_4_·H_2_O, which was continuously bubbled with 5% CO_2_ and 95% oxygen. The artery was carefully cleaned of blood, fat and loose connective tissues, then cut into ∼2–3 mm length rings under a stereo microscope (Nikon SMZ-2T, Tokyo, Japan). One side of the artery ring was connected to a stainless steel hook fixed at the bottom of the organ bath chamber and the other side was connected to a stainless steel hook connected to a tension transducer (52-9503; Harvard Bioscience, Holliston, MA). Each aortic ring was entirely submerged in a water-jacketed organ bath chamber containing 20 mL PSS, maintained at a constant temperature of 37 °C. A stable baseline tension of 1.0 g was applied to vessel rings, which produced a maximum contraction in response to 60 mM KCl. Rings were then allowed to equilibrate in the organ chamber for 60 min, with changes of PSS at 30 min intervals. Contractile responsiveness of aortic rings was stabilized by exposure to 60 mM KCl followed by two exposures to 1 μM phenylephrine (Phe) to precontract rings prior to commencing the experimental protocol. During vessel isolation and preparation of rings for endothelial-dependent experiments, great care was taken not to damage the endothelial layer. For endothelium-denuded experiments, the endothelial layer was mechanically removed by gently rubbing the luminal surface of the aortic rings with fine forceps. The intactness or appropriate removal of the endothelium was confirmed by the presence or absence of vascular relaxation, respectively, following treatment with carbachol (10 μM).

### Cell culture and transfection

Human embryonic kidney 293 (HEK293) cells were cultured at 37 °C and 5% CO_2_ in Dulbecco’s Modified Eagle Medium (11995-065; Life Technologies, Camarillo, CA) supplemented with 1× antibiotic–antimycotic reagent (15240; Life Technologies, Camarillo, CA) and 10% foetal calf serum. Yellow fluorescent protein-tagged hTRPC3 (NCBI Reference Sequence: NP_003296.1), cloned into the pcDNA3 vector, and the M3 muscarinic acetylcholine receptor, cloned into the pRK5-HA vector, were transiently co-transfected into HEK293 cells at a 1:1 ratio (0.5 µg each) using Lipofectamine 2000 Transfection Reagent (Life Technologies, Camarillo, CA), as recommended by the vendor. In brief, 1 µg DNA and 5 µL transfection reagent were separately and thoroughly mixed in 50 µL and 45 µL Opti-MEM (31985-070; Life Technologies, Camarillo, CA), respectively, and incubated at room temperature for 5 min. Thereafter, the two solutions were mixed and incubated at room temperature for 20 min before adding to 90% confluent HEK293 cells growing on 12-well plates in Opti-MEM media (antibiotic- and serum-free). Cells were harvested and plated on cover slips 24 h after transfection and subjected to whole-cell patch-clamp experiments.

### Electrophysiology and solutions

Patch pipettes were pulled from thin-wall filament glass capillaries (GC 150TF-7.5; Harvard Apparatus, Holliston, MA) to a resistance of 3–4 MΩ using a vertical pipette puller (PC-10; Narishige Group Products, Amityville, NY). Transfected cells, characterized by their yellow fluorescence following illumination at 514 nm, were identified using an inverted microscope (ECLIPSE Ti; Nikon, Tokyo, Japan). Whole-cell voltage-clamp experiments were performed at room temperature using an Axopatch 200B capacitor-feedback patch-clamp amplifier (Molecular Devices, Sunnyvale, CA) connected to a Digidata-1440A Digitizer (Molecular Devices, Sunnyvale, CA). Currents were recorded during application of a linear voltage-ramp protocol (−100 to +100 mV; holding potential, −60 mV). The standard extracellular solution contained 140 mM NaCl, 5 mM KCl, 10 mM HEPES and 0.5 mM EGTA, titrated to pH 7.4 with NaOH. The standard intracellular solution contained 140 mM CsCl, 3 mM MgCl_2_, 1 mM ATP, 1.5 mM CaCl_2_, 10 mM HEPES and 5 mM EGTA, titrated to pH 7.2 with CsOH. For analysis of TRPC3 currents, carbachol (100 µM) was added to the perfusate to activate the muscarinic receptor.

### Chemicals and reagents

All chemicals were purchased from Sigma-Aldrich, Yongin-si, Korea, unless otherwise noted. PCE was reconstituted in DMSO at a concentration of 1000 μg/mL.

### Statistical analysis

All data are shown as means ± standard error of mean (SEM), and differences between conditions were analysed using an unpaired Student’s *t*-test and two-way analysis of variance (ANOVA). Concentration–response relationships were fitted to a sigmoidal curve using the built-in function in Origin pro 8.1 (OriginLab Co., Northampton, MA), and analysed by two-way ANOVA followed by Student’s *t*-test using the same software. A *p* value <0.05 was considered statistically significant.

## Results

### HPLC analysis of PCE

The newly established HPLC-PDA method was used for the simultaneous determination of the eight marker compounds in PCE. All analytes eluted within 30 min, and the typical HPLC chromatogram of standard solution and 70% ethanol extract of *P. corylifolia* are shown in Figure 1(A,B). Samples were quantified using a PDA detector based on their *λ*_max_ in the UV spectrum, as follows: bakuchiol 260 nm, bavachin and bavachinin 275 nm, psoralen 290 nm, isopsoralen 300 nm, psoralidin 345 nm and isobavachalcone 370 nm. The correlation coefficients (*r*^2^) of calibration curves for the eight compounds were all greater than 0.999. Under optimized HPLC chromatography conditions, the amounts of the eight marker compounds in PCE were as follows: psoralen 32.44 ± 0.06 mg/g, isopsoralen 30.92 ± 0.08 mg/g, bavachin 9.67 ± 0.11 mg/g, corylin 4.30 ± 0.04 mg/g, psoralidin 5.88 ± 0.02 mg/g, isobavachalcone 10.42 ± 0.02 mg/g, bavachinin 15.21 ± 0.04 mg/g and bakuchiol 391.32 ± 1.90 mg/g.

### Effects of PCE on phenylephrine-induced contraction of endothelium-intact and -denuded aortic rings

**Figure 2.**
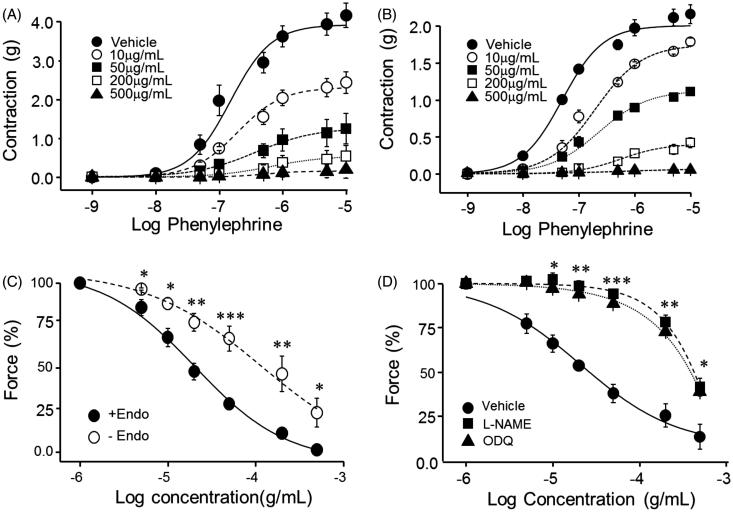
Endothelium dependence of PCE-induced vasorelaxation of Phe-contracted, isolated rat aortic rings. (A, B) Different concentrations of PCE were added to endothelium-intact (A) or -denuded (B) aortic rings 30 min prior to performing cumulative Phe concentration–response studies. (C) PCE concentration dependent relaxation was measured in endothelium-intact and -denuded rat aortic rings pre-contracted with 10 µM Phe. (D) Effects of eNOS and GC inhibition on PCE-induced vasorelaxation of Phe-pre-contracted rat aortic rings. Rings were pretreated for 30 min with the non-specific NOS inhibitor, l-NAME (50 µM) or the GC inhibitor ODQ (20 µM) prior to pre-contracting with Phe. Data are means ± SEM of the relaxing effect, expressed as a percentage of the maximum Phe contraction (*n* = 6–8; *p < 0.05, **p < 0.01, ***p < 0.001).

Treatment of endothelium-intact aortic rings with different concentrations of PCE (10, 50, 200 or 500 μg/mL) reduced the maximal contraction induced by different concentrations of Phe (10^−9^ to 10^−5^ M), exhibiting a 50% inhibitory concentration (IC_50_) of 12.45 μg/mL at a Phe concentration of 10 μM (*n* = 6–8; Figure 2(A)). Maximal relaxation was achieved at a PCE concentration of 500 μg/mL, which reduced maximal Phe-induced contraction by 95.96 ± 2.13%. As shown in Figure 2(B), the relaxation-promoting effect of PCE was diminished in endothelium-denuded arterial rings, as reflected in the approximately five-fold higher IC_50_ value (60.27 μg/mL) at the same concentration of Phe (10 μM; *n* = 6–8).

To further investigate the endothelium dependence and independence of PCE action, we precontracted endothelium-intact and -denuded aortic rings with a fixed concentration of Phe (10 μM), and then treated them with different concentrations of PCE (Figure 2(C)). Under endothelium-intact conditions, the IC_50_ value for PCE was 61.27 ± 3.11 μg/mL (*n* = 6–8). The vasorelaxing effect of PCE was significantly attenuated in the absence of a functional endothelium, as evidenced by an increase in IC_50_ (211.13 ± 18.74 μg/mL, *n* = 6–8; *p* < 0.001 compared to endothelium-intact artery at 50 μg/mL PCE). However, removal of the endothelium did not totally abolish the vasorelaxing activity of PCE. These results indicate that the vasodilatory effects of PCE reflect both endothelium-dependent and -independent mechanisms.

### Effect of inhibiting NO/cGMP on PCE-induced vasorelaxation

To evaluate the involvement of NO in PCE-induced vasorelaxation, we investigated the effects of inhibiting nitric oxide synthase (NOS) with the nonselective blocker, N^ω^-nitro-l-arginine methyl ester hydrochloride (l-NAME), and blocking soluble guanylate cyclase (sGC) with the selective blocker, 1H-[1,2,4]oxadiazolo[4,3-a]quinoxalin-1-one (ODQ). As shown in Figure 2(D), Pretreatment with l-NAME (50 µM) or ODQ (20 µM) considerably reduced the relaxation effect of PCE (*n* = 6–8, *p* < 0.001 at 50 μg/mL PCE). These results suggest that endothelial NOS (eNOS) and sGC make a profound contribution to the relaxation effect of PCE.

### Effect of K^+^ channel blockade on PCE-induced vasorelaxation

**Figure 3.**
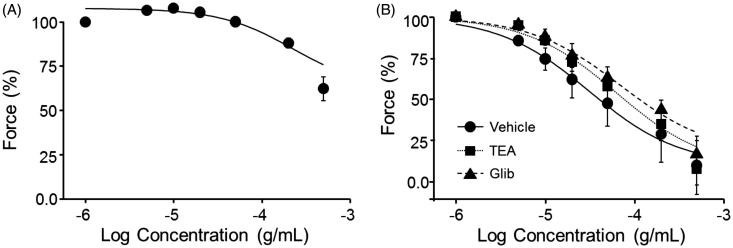
The role of K ^+^ channels in PCE actions. (A) Effect of PCE on high K^+^ (60 mM)-pre-contracted, isolated rat aortic rings. (B) Effects of inhibitors of K_Ca_ channels (TEA) and KATP channels (glibenclamide) on PCE-induced relaxation of isolated aortic rings. Cumulative concentration–response curves for PCE following a 30-min incubation with vehicle (*n* = 4), 1 mM TEA (*n* = 6) or glibenclamide (*n* = 6) in aortic rings precontracted with 10 µM Phe are shown. Data are means ± SEM of the relaxing effect, expressed as a percentage of the maximum Phe contraction.

To determine the role of K^+^ channels in the PCE-induced vasorelaxation mechanism, we measured the concentration-dependent relaxant effect of PCE on KCl-precontracted aortic rings. PCE did not cause a measurable relaxation of KCl-precontracted rings (Figure 3(A)). Pretreatment with 1 mM tetraethylammonium (TEA), a nonselective Ca^2+^-activated K^+^ (K_Ca_) channel blocker, or glibenclamide (5 µM), a selective inhibitor of ATP-sensitive K^+^ (K_ATP_) channels, also failed to modulate PCE-induced relaxant effects. Collectively, these results indicate that K^+^ channels make no appreciable contribution to PCE-induced vasorelaxation activity (Figure 3(B)).

### Effect of blocking Ca^2+^-permeable channels on PCE-induced vasorelaxation

**Figure 4.**
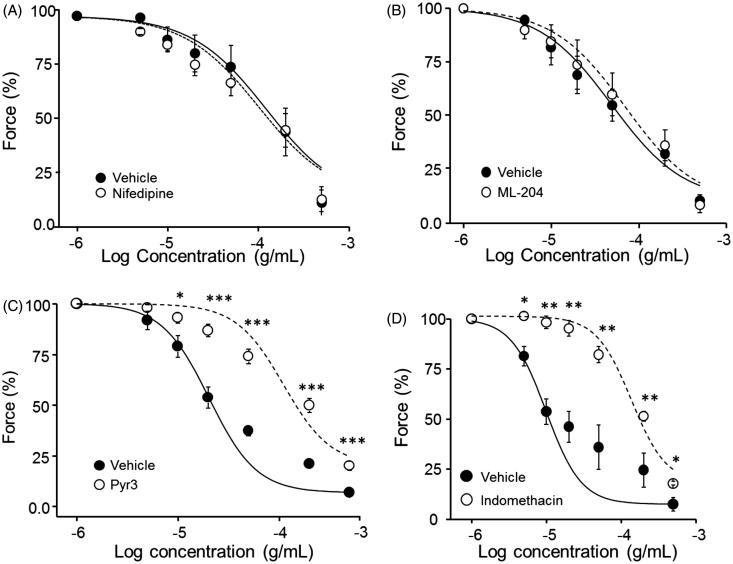
Effects of PCE on Ca^2+^ entry. The effects of nifedipine (A), ML-204 (B), Pyr3 (C) and indomethacin (D) were tested. Aortic rings were pre-incubated with each chemicals for 30 min, and relaxation of Phe (10 µM)-pre-contracted rings by different concentrations of PCE was measured. Data are means ± SEM of the relaxing effect, expressed as a percentage of the maximum Phe contraction (*n* = 5–6; *p < 0.05, **p < 0.01, ***p < 0.001).

To further define the possible role of voltage-dependent and -independent Ca^2+^ permeable channels in the PCE vasodilatory activity, we incubated endothelium-intact aortic rings with the L-type VDCC blocker, nifedipine (1 µM). As shown in Figure 4(A), inhibition of VDCCs did not affect vasodilatory responses, suggesting little or no involvement of VDCCs in the PCE vasorelaxation mechanism. To assess whether PCE affects non-voltage-gated Ca^2+^ entry or store-operated Ca^2+^ channels, we pretreated endothelium-intact aortic rings with the TRPC4-selective inhibitor, ML-204 (10 μM), and the TRPC3-selective inhibitor, pyrazole-3 (Pyr3) (10 μM). As shown in Figure 4(C,D), only Pyr3 significantly decreased the vasodilatory effect of PCE on rings precontracted with Phe, increasing the IC_50_ for PCE from 24.95 ± 5.41 μg/mL (vehicle control) to 160.67 ± 81.77 μg/mL (*n* = 5–6; *p* < 0.001 at 50 μg/mL PCE). These results suggest that receptor-operated Ca^2+^ entry, particularly TRPC3-dependent Ca^2+^ entry, may play a prominent role in the vasodilatory effect of PCE on rat aortic rings.

### Role of prostacyclin production in PCE-induced vasorelaxation

To assess the role of the cyclooxygenase (COX) product, prostacyclin (PGI_2_), in mediating the vasodilatory action of PCE, we inhibited COX with indomethacin (10 μM). Pretreatment of aortic rings with indomethacin inhibited PCE-induced vasorelaxation, increasing the IC_50_ for PCE from 34.35 ± 16.47 μg/mL (vehicle) to 185.54 ± 21.08 μg/mL (*n* = 5–6; *p* < 0.05 at 50 μg/mL PCE; Figure 4(D)). These findings suggest the possible involvement of endothelium-derived COX products in the vasodilatory action of PCE.

### Effects of PCE on TRPC3 channel-mediated currents

**Figure 5.**
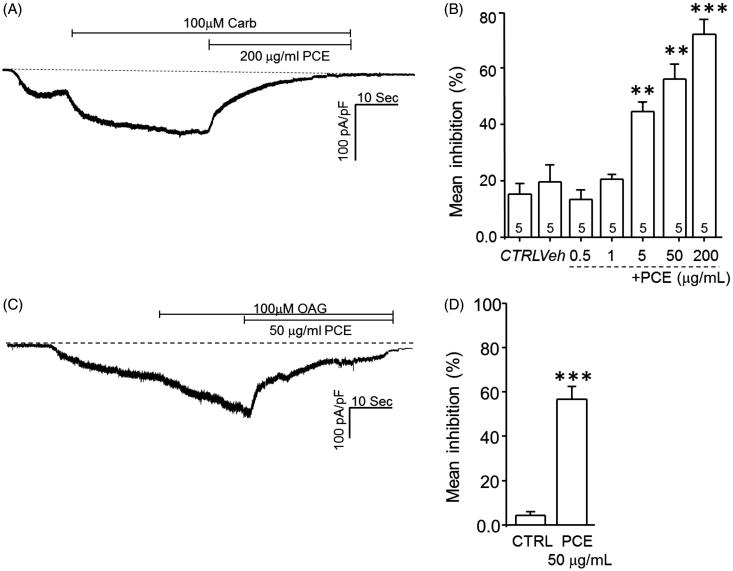
Effects of PCE on TRPC3 currents in HEK293 cells overexpressing M3 muscarinic receptors and human TRPC3 channels. Transiently transfected HEK293 cells were stimulated with 100 µM carbachol (A, B) or 100 µM OAG (C, D), followed by addition of 200 µg/mL or 50 µg/mL PCE, respectively. Representative traces showing the inhibitory effects of 200 or 50 µg/mL PCE on carbachol-stimulated (A) and OAG stimulated hTRPC3 (C) currents, respectively. Effects of carbachol and OAG are summarized in B and D, respectively. Bars represent the mean values of inhibition ± SEM (**p < 0.01, ***p < 0.001).

To more directly evaluate the involvement of Ca^2+^-permeable TRPC3 channels, we assessed the effects of PCE treatment on receptor-stimulated TRPC3 ionic currents using an electrophysiological approach. To this end, we transiently overexpressed the M3 muscarinic receptor (M3R) and hTRPC3 in HEK293 cells. As shown in Figure 5(A,B), 200 μg/mL PCE inhibited M3R-stimulated hTRPC3 currents by 89.12 ± 1.01% (*p* < 0.001, *n* = 5–6); this inhibition was concentration dependent. We also investigated the effects of PCE on hTRPC3 channel activity following direct stimulation with the endogenous diacylglycerol (DAG) analogue, OAG (1-oleoyl-2-acetyl-sn-glycerol). PCE (50 μg/mL) also inhibited OAG (100 μM)-activated TRPC3 currents by 56.71 ± 5.77% (*p* < 0.001, *n* = 5–6; Figure 5(C,D)). These results suggest that TRPC3 channels are directly involved in mediating the vasodilatory effects of PCE on rat aortic rings.

### Effects of constituents of PCE on TRPC3 channel-mediated currents

**Figure 6.**
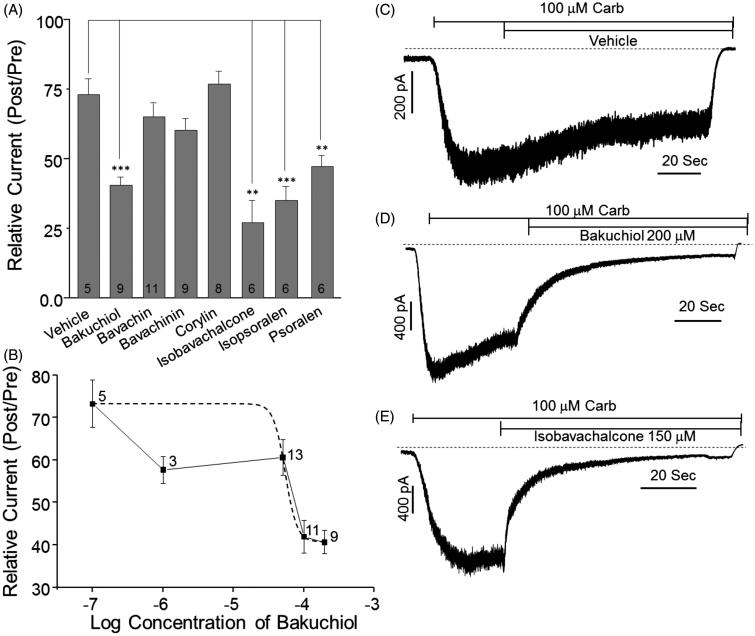
Effects of constituents of PCE on TRPC3 currents in HEK293 cells. (A) HEK293 cells transiently transfected with TRPC3 were stimulated with 100 µM carbachol followed by addition of 200 µg/mL or 150 µg/mL of the indicated chemical constituent. (B) The concentration-dependent inhibition of TRPC3 currents by bakuchiol was studied. (C–E) Representative traces for treatment with vehicle (C), bakuchiol (D) and isobavachalcone (E) are shown. Bars represent the mean values of inhibition ± SEM (**p < 0.01, ***p < 0.001).

As shown in Figure 1, numerous constituents of PCE have been identified, including bakuchiol and bavachin, among others. Therefore, we briefly evaluated the effects of individual PCE components on TRPC3 currents in TRPC3 channel-overexpressing HEK293 cells. Interestingly, bakuchiol, isobavachalcone, isopsoralen and psoralen significantly inhibited TRPC3 currents (Figure 6(A,C,D,E). The concentration-dependent action of one of these effective chemicals, bakuchiol, was further studied, revealing an IC_50_ value of 54.5 μM (Figure 6(B)).

### Effects of constituents of PCE on phenylephrine-induced contraction of endothelium-intact aortic rings

**Figure 7.**
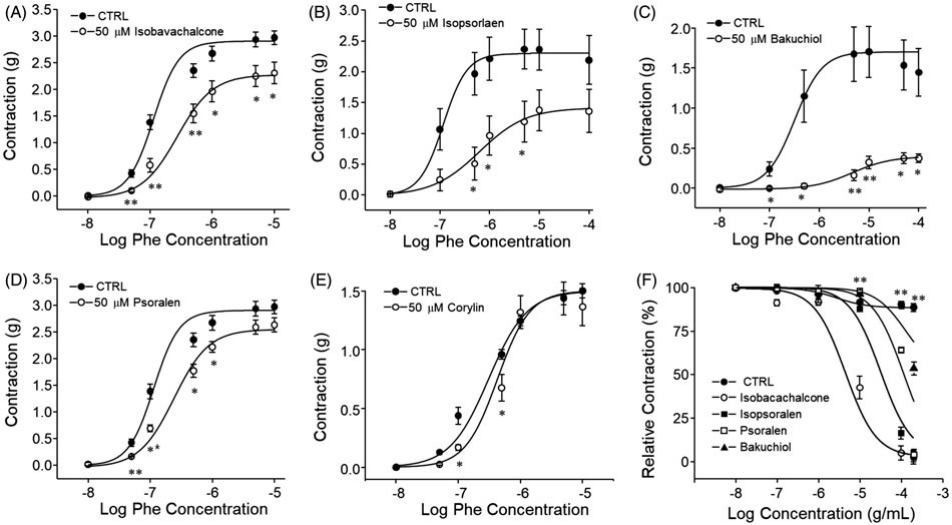
Effects of constituents of PCE on Phe-induced contraction of endothelium-intact aortic rings. Each constituent of PCE (final concentration, 50 µM) was added to endothelium-intact aortic rings 30 min prior to performing cumulative Phe concentration–response studies. (A–E) Representative concentration–response curve of isobavachalcone (A), isopsoralen (B), bakuchiol (C), psoralen (D) and corylin (E) are shown. (F) Effects of accumulative treatment with constituents on Phe (1 µM)-induced aortic contraction. Bars represent the mean values of inhibition ± SEM (*n* = 5). *p < 0.05, **p < 0.01 versus control.

As shown in Figure 6, four constituents of PCE (isobavachalcone, bakuchiol, isopsoralen and psoralen) strongly inhibited TRPC3 currents measured in HEK293 cells heterogeneously expressing hTRPC3. To assess the effects of individual PCE components on Phe-induced contraction of aortic rings, we pretreated rat aortic rings with each of the five chemicals for 30 min prior to measuring Phe dose responses. Bakuchiol, isobavachalcone, isopsoralen and psoralen significantly reduced Phe-induced vasoconstriction in a concentration dependent manner, whereas corylin, which lacked inhibitory effects on TRPC3 currents, had no significant effect on aortic contraction (Figure 7(A–E)). The rank order of inhibitory actions of each constituents followed the order of inhibition of TRPC3 currents.

## Discussion

The results of the present study indicate that PCE exerts a vasorelaxing effect on isolated rat aortic rings and identified a number of associated modulatory mechanisms. Our findings indicate that PCE-induced vasodilation was strongly dependent on the presence of the endothelium as well as COX products and Ca^2+^ entry through canonical TRPC3 channels. Contrary to a previous report on bakuchicin, which causes relaxation in vascular smooth muscle via eNOS together with blockade of VDCCs, our current results demonstrate the involvement of nonselective cation channels (Li et al. 2011). PCE targets TRPC3-mediated Ca^2+^ entry rather than VDCC-mediated Ca^2+^ entry, and the resulting relaxing effect of PCE is partially blocked by indomethacin. The following eight compounds in PCE were identified: psoralen, isopsoralen, bavachin, corylin, psoralidin, isobavachalcone, bavachinin and bakuchiol. Of these, bakuchiol and isopsoralen were found to be the most effective in causing relaxation of Phe-induced contraction.

*P. corylifolia* is among traditional herbal compounds that have been widely used for the treatment of skin disease, inflammatory disease and tuberculosis, among other ailments (Chopra et al. 2013). Phytochemical studies have demonstrated that compounds such as coumarins, flavonoids, phenolic monoterpenes and volatile oils are abundant in *P. corylifolia* (Ruan et al. 2007). Among the various chemical and pharmacologic constituents identified in our extract of *P. corylifolia* were bakuchiol, psoralen, isopsoralen, bavachinin, isobavachalcone, bavachin, psoralidin and corylin. These constituents have been intensively studied for their biological activity (Chaudhuri and Bojanowski 2014). Bakuchiol from an ethanol extract of *P. corylifolia* seeds has been reported to be an antibacterial agent (Hsu et al. 2009); psoralen has been shown to possess anticancer activity, and isopsoralen (Guo et al. 2003; Wang et al. 2011) and bakuchiol from ethanol extracts of *P. corylifolia* enhance cytotoxicity towards tumours (Bapat et al. 2005). Among the various biological actions of PCE ingredients is inhibition of platelet aggregation, an effect that has been attributed to isobavachalcone (Matsuda et al. 2009). Bakuchiol, coryliforlin and corylin from diverse extracts of *P. corylifolia* also show strong antioxidant activity (Haraguchi et al. 2000). Although multiple biological activities of *P. corylifolia* have been reported, there is little information regarding the vascular actions of *P. corylifolia*. Among the compounds isolated from *P. corylifolia*, only bakuchicin, which acts in an endothelium-dependent manner via the NO/cGMP pathway, has been shown to have a vasorelaxing effect (Li et al. 2011). In this latter study, Li et al. demonstrated that bakuchicin induced an increase in NO production that resulted in VDCC inhibition and caused relaxation of smooth muscle. However, our current study revealed that bakuchiol, the most abundant chemical in PCE, strongly inhibited TRPC3-mediated ionic currents, whereas bavachinin did not, indicating that these two different components of PCE exert their vascular actions via different mechanisms.

NO released from endothelial cells regulates vascular tone and blood pressure in response to vasodilatory stimuli, such as acetylcholine, bradykinin and shear stress (Arnal et al. 1999; Filosa et al. 2013). These NO-releasing stimuli are involved in several physiological processes, including thrombosis formation, vascular remodelling and smooth muscle proliferation. Upon stimulation, increases in intracellular Ca^2+^ initiate Ca^2+^/calmodulin complex-dependent induction of eNOS, which produces the diffusible vasorelaxing factor, NO. Gaseous transmission of NO activates GC isoenzymes in smooth muscle, raising cGMP levels and ultimately leading to vasodilation (Waldman et al. 1988). In the current study, we found that removal of the endothelium greatly attenuated PCE-induced relaxation, as did nonselective blockade of NOS and sGC with l-NAME and ODQ, respectively. This implies that the NO/cGMP pathway plays a crucial role in the vasorelaxation activity of PCE. Vasoactive prostanoids, such as the prostaglandins, PGD_2_, PGE_2_ and PGF_2α_, prostacyclin (PGI_2_) and thromboxane A_2_ (TXA_2_), are produced by the sequential actions of COX and specific prostanoid synthases (Vanhoutte 2009). In the present study, inhibiting the COX pathway with indomethacin significantly reduced PCE-induced relaxation, implying a role for COX-derived products in the vasodilatory action of PCE.

It is well established that discrete Ca^2+^ transients in endothelial cells emerging from both internal stores and plasmalemmal cation channels couple to specific membrane K^+^ channels, promoting endothelial hyperpolarization (Qian et al. 2014). Hyperpolarization of the membrane potential of underlying smooth muscle decreases intracellular Ca^2+^ concentration by reducing the driving force for Ca^2+^ through VDCCs (Nelson et al. 1995). We found that inhibition of L-type VDCCs only modestly reduced the vasodilatory activity of PCE, suggesting that L-type VDCCs are not the main target of the vasorelaxation activity of PCE in the rat aorta. K^+^ channels are also among the dominant ion-conduction pathways in endothelial and vascular smooth muscle cells (Baranowska et al. 2007), and their activity contributes to determining and regulating membrane potential and vascular tone. However, our experiments showed that contractions induced by 60 mM KCl were not measurably affected by PCE treatment, and pretreatment with the nonselective K_Ca_ channel blocker, TEA, or the K_ATP_ channel blocker, glibenclamide, did not attenuate the relaxant response of aortic tissues to PCE. These results suggest that PCE-induced relaxation of vascular smooth muscle is independent of K^+^ channels.

In addition to VDCCs, TRPC channels are important in mediating Ca^2+^ entry during excitation-contraction coupling in smooth muscle cells; they are also involved in receptor-operated Ca^2+^ entry in endothelial cells (Brayden et al. 2008; Zhang and Gutterman 2011). A number of Ca^2+^-permeable TRPC channels have been identified in vascular smooth muscle cells and endothelial cells. Freichel et al. (2001) demonstrated a critical role for TRPC4 channels in store-operated Ca^2+^ entry in mouse endothelial cells. Kochukov et al. (2013) reported that both TRPC1 and TRPC3 participate in endothelial cell Ca^2+^ influx and vasorelaxation of the aorta. Inoue et al. (2001) also provided heterologous expression data for TRPC6 in smooth muscle cells, demonstrating a role for TRP channels in smooth muscle cell receptor-operated Ca^2+^ entry in the portal vein. We showed that inhibition of TRPC3 with its specific blocker, Pyr3, strongly attenuated PCE-induced vasorelaxation. These findings support a previous report that TRPC3-mediated Ca^2+^ influx leads to smooth muscle contraction and thus vasocontraction (Yeon et al. 2014), and is consistent with the observed operation of both endothelium-dependent and -independent mechanisms in the vasodilatory action of PCE. Although bakuchicin has been reported as a vasoactive constituent of *P. corylifolia*, our data suggest that PCE contains several vasoactive chemicals, including bakuchiol, isobavachalcone, isopsoralen and psoralen, that inhibit Ca^2+^ influx through TRPC3 channels, decrease intracellular Ca^2+^ and cause relaxation. These compounds significantly attenuated the Phe-induced contraction; thus, their vasorelaxing effects are likely attributable to an inhibition of calcium entry through TRPC3 channels.

## Conclusions

The present study revealed that PCE acts predominantly through endothelium-dependent vasodilation mediated by the NO/cGMP pathway. Additionally, the COX-dependent pathway and nonselective cation channels, possibly TRPC3, are involved in the PCE-induced vasorelaxation effect. Several constituents of PCE, including bakuchiol, isobavachalcone, isopsoralen and psoralen, modulate TRPC3-mediated ionic currents, and therefore may be responsible for the vasoactive effects of PCE. The major active compounds in PCE appeared to be bakuchiol, which is also the most abundant compound in the ethanol extract; the other compounds present likely synergize with bakuchiol to enhance the vascular smooth muscle-relaxing effects of PCE. Additional *in vivo* studies will ultimately benefit the development of PCE-based antihypertension therapeutics.
